# Overexpression of PGC‐1α in aging muscle enhances a subset of young‐like molecular patterns

**DOI:** 10.1111/acel.12707

**Published:** 2018-02-10

**Authors:** Sofia Garcia, Nadee Nissanka, Edson A. Mareco, Susana Rossi, Susana Peralta, Francisca Diaz, Richard L. Rotundo, Robson F. Carvalho, Carlos T. Moraes

**Affiliations:** ^1^ Department of Neurology University of Miami Miller School of Medicine Miami FL USA; ^2^ Neuroscience Graduate Program University of Miami Miller School of Medicine Miami FL USA; ^3^ Graduate Program in Environment and Regional Development University of Western São Paulo Presidente Prudente Brazil; ^4^ Department of Cell Biology University of Miami Miller School of Medicine Miami FL USA; ^5^ Institute of Biosciences São Paulo State University (UNESP) Botucatu Brazil

**Keywords:** aging, lifespan, longevity, mouse models, mitochondria, skeletal muscle

## Abstract

PGC‐1α is a transcriptional co‐activator known as the master regulator of mitochondrial biogenesis. Its control of metabolism has been suggested to exert critical influence in the aging process. We have aged mice overexpressing PGC‐1α in skeletal muscle to determine whether the transcriptional changes reflected a pattern of expression observed in younger muscle. Analyses of muscle proteins showed that Pax7 and several autophagy markers were increased. In general, the steady‐state levels of several muscle proteins resembled that of muscle from young mice. Age‐related mtDNA deletion levels were not increased by the PGC‐1α‐associated increase in mitochondrial biogenesis. Accordingly, age‐related changes in the neuromuscular junction were minimized by PGC‐1α overexpression. RNA‐Seq showed that several genes overexpressed in the aged PGC‐1α transgenic are expressed at higher levels in young when compared to aged skeletal muscle. As expected, there was increased expression of genes associated with energy metabolism but also of pathways associated with muscle integrity and regeneration. We also found that PGC‐1α overexpression had a mild but significant effect on longevity. Taken together, overexpression of PGC‐1α in aged muscle led to molecular changes that resemble the patterns observed in skeletal muscle from younger mice.

## 1 INTRODUCTION

Mitochondrial biogenesis, which depends on two genomes (nuclear and mitochondrial), is regulated primarily by the peroxisome proliferator‐activated receptor γ family of transcriptional co‐activators (PGC‐1 family). As transcription co‐activators, they do not interact directly with DNA, but rather with transcription factors. These transcription factors include the nuclear respiratory factors 1 and 2 (NRF‐1 and NRF‐2), the estrogen‐related receptor alpha (ERRα) and the PPAR family of transcription factors (Scarpulla, Vega, & Kelly, 2012). The activation of these transcription factors leads to an increase in the expression of several mitochondrial proteins, including the mitochondrial transcription factor A (TFAM) and B2 (TFB2M) (Scarpulla et al., 2012). TFAM and TFB2M are essential for mtDNA replication, transcription, and maintenance. By regulating the activity of these transcription factors, PGC‐1 co‐activators are able to influence the expression of mitochondrial proteins, both encoded by the mtDNA and the nuclear DNA. In addition to regulating mitochondrial biogenesis, PGC‐1 co‐activators are involved in modulating other metabolic pathways including fatty acid oxidation, lipogenesis, gluconeogenesis, and thermogenesis.

In skeletal muscle, PGC‐1α also has been shown to regulate skeletal muscle fiber‐type switch, glucose transport, and lipid utilization (Kupr & Handschin, 2015). PGC‐1α knockout (KO) mice have a lower ratio of oxidative to glycolytic muscle fibers. The PGC‐1α muscle KO animals have reduced endurance capacity, increased fiber damage, and elevated markers of inflammation following treadmill running (Kupr & Handschin, 2015). PGC‐1α also regulates mitochondrial antioxidant factors. Reduced mRNA levels of SOD1, SOD2, and GPx1 were observed in skeletal muscle from PGC‐1α KO mice. On the other hand, PGC‐1α overexpression mice showed an upregulation of SOD2 protein in skeletal muscle (Kupr & Handschin, 2015). The inflammatory process is also regulated by PGC‐1α, through mechanisms that are not well understood. PGC‐1α regulation of the antioxidant defense may play a role in inflammation, as oxidative stress can induce an inflammatory response through activation of the redox‐sensitive nuclear factor kappa B (NF‐κB; Kupr & Handschin, 2015).

Chronic diseases and aging show common patterns in several pathways, including alterations in inflammatory, and metabolic patterns as well as oxidative stress, insulin resistance, genomic instability, DNA damage, and dysfunction of mitochondria and telomeres (Xiong, Patrushev, Forouzandeh, Hilenski, & Alexander, 2015). PGC‐1α modulates most of these processes and therefore has been implicated as a key factor in the aging process (Dillon, Rebelo, & Moraes, 2012).

In the present study, we analyzed the consequence of overexpression of PGC‐1α in aging skeletal muscle and found that it adapted the aging muscle to molecular patterns that resemble younger muscle.

## 2 RESULTS

### 2.1 Aged muscle overexpressing PGC‐1α has more oxidative fibers, OXPHOS proteins, autophagy markers, and less 20S proteasome

To obtain insights on the effect of PGC‐1α overexpression in very old mice, we allowed C57/BL6J and C57/BL6J overexpressing PGC‐1α in muscle to age to natural death. We sacrificed four extremely old mice from each group (between 29 and 34 months, Table S1) for molecular studies. Although these mice were a mix of males and females, the analyzed changes triggered by overexpression of PGC‐1α in muscle (described below) were robust enough to minimize sex‐specific differences. Therefore, males and females were analyzed together.

**Figure 1:**
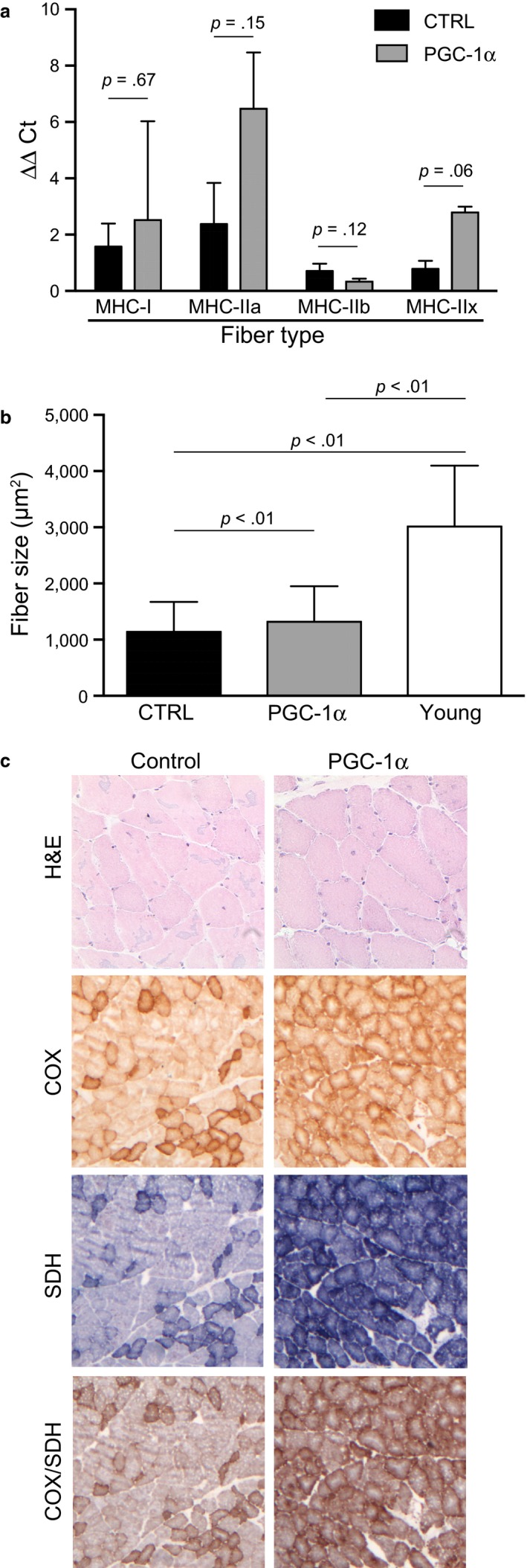
Fiber‐type composition of aged mice overexpressing PGC‐1α in muscle. Panel a shows the analysis of MHC isotypes. There was an increase in slow fiber type (more oxidative) in the transgenic mice. *N* = 4 per group. Fiber diameter size was determined by automated scanning of >1,300 fibers after immunostaining for laminin. Young muscle (4 months old) was included in these analyses (Panel b). Histological staining for H&E and enzyme activity of mitochondrial cytochrome oxidase (COX) and succinate dehydrogenase (SDH) in muscle are shown in panel c

We isolated quadriceps, heart, and brain from these mice for further studies. Fiber typing was determined in quadriceps by quantitative RT‐qPCR using primers specific for the different myosin heavy‐chain isoforms. As expected, there was a trend toward an increase in oxidative fibers in the aged PGC‐1α transgenic mice compared to aged controls (Figure 1a). Next, we stained muscle sections with laminin to visualize the sarcolemma and determine the area of > 1,300 fibers. These analyses showed that the aged PGC‐1α muscle had muscle fibers that were slightly but significantly larger than in the aged controls. Confirming previous findings, young (3 months old) mice had markedly larger fibers than aged mice (Figure 1b).

Histological analyses by H&E showed no major abnormalities in the quadriceps, with a few fibers in aged mice containing central nuclei in both groups. Staining for activities of mitochondrial OXPHOS enzymes cytochrome *c* oxidase (COX) and succinate dehydrogenase (SDH) showed marked increases in the PGC‐1α group, as expected (Figure 1c). We also performed these analyses in heart, which has been shown to have a smaller fold increase in the MCK‐PGC‐1α transgene expression compared to skeletal muscle (Lin et al., 2002). However, we found no obvious difference in the staining intensity for COX, SDH, or the combined staining in heart (not shown). Because metabolic changes in muscle could have a systemic effect, we also studied the CNS of these mice by H&E staining and immunostaining for NeuN and GFAP. We did not detect any obvious differences in cell numbers or gross pathology between the two aged groups (not shown).

**Figure 2:**
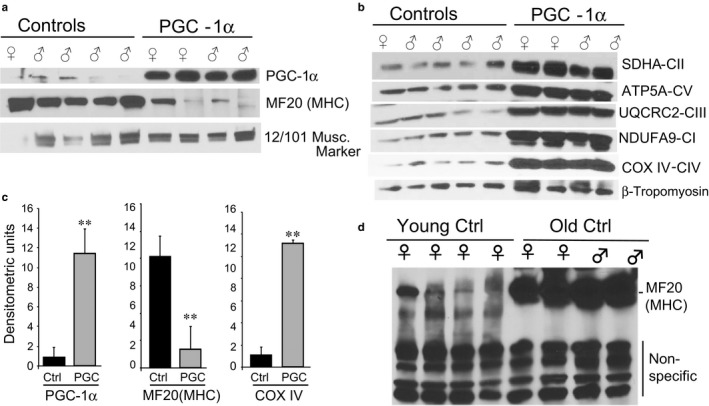
Western blot analyses of skeletal muscle proteins. Panel a shows the levels of PGC‐1α and two muscle markers from the DSB repository (myosine heavy‐chain MF20 and 12/101 marker). Panel b shows the levels of OXPHOS proteins, namely: SDHA, ATP5A, UQCRC2, NDUFA9, and COX IV. Panel c shows the quantification of selected blots in panels a and b. **p < .05

**Figure 3:**
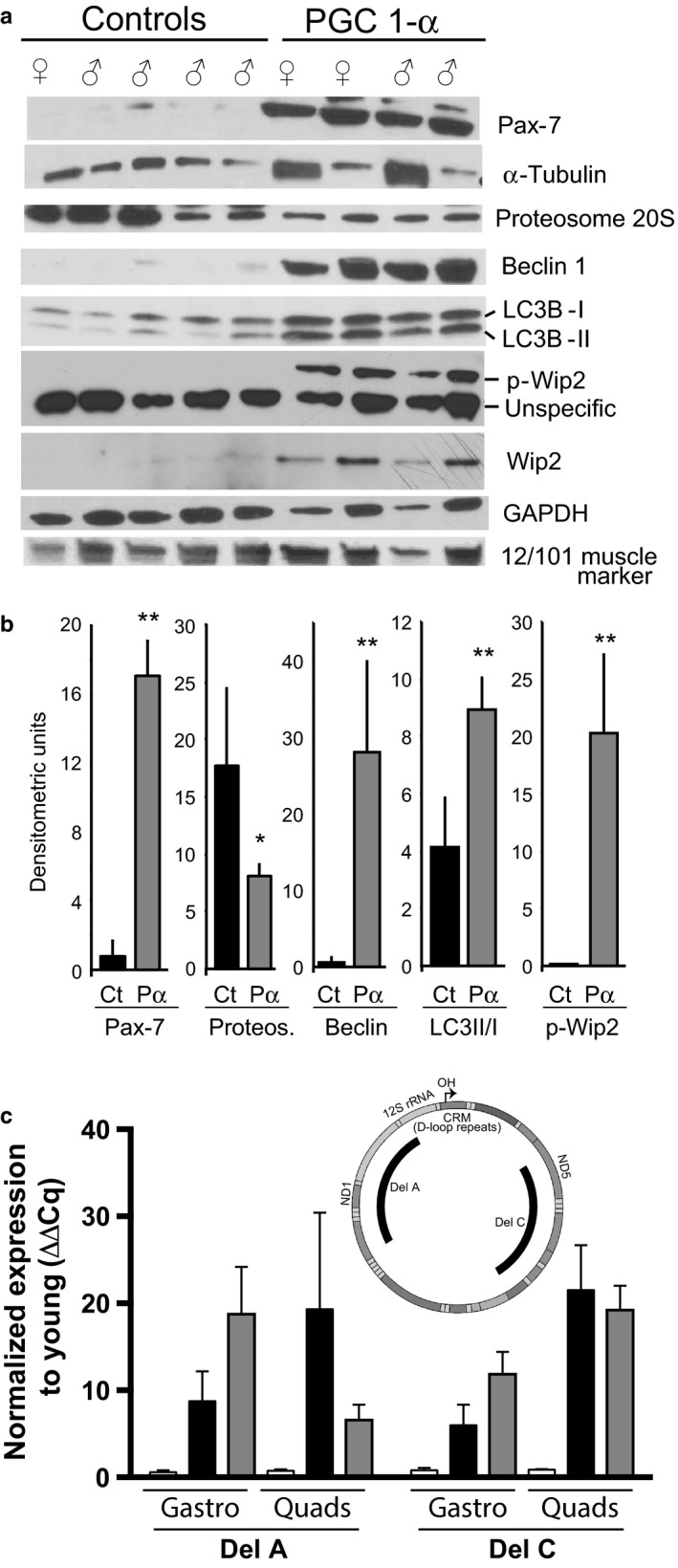
Analyses of muscle proteins associated with muscle wasting. Panel a shows Western blots of muscle proteins showing marked alterations in steady‐state levels. These include Pax7 (satellite cell marker), proteasome 20S (proteome degradation), beclin (autophagy), LC3II/I ratio (autophagy), wip2, and p‐wip2 (autophagy). Loading controls are also indicated (α‐tubulin, p‐wip2‐unspecific band, and GAPDH). Panel b shows the quantification of protein of interest. **p < .05. Panel c shows the quantification of previously reported age‐related mtDNA deletions (known as Deletion A and Deletion C). These were increased in aged muscle (measured by qPCR), but no different between the groups

Skeletal muscle was further characterized by analyzing the levels of PGC‐1α by Western blot. The aged transgenic mice had approximately 10 times the levels of PGC‐1α compared to aged controls (Figure 2a). Interestingly, a band detected by a monoclonal antibody reported to detect all muscle myosin isoforms (MF20 from the Developmental Studies Hybridoma Bank—DSHB) was markedly decreased in the aged PGC‐1α muscle, a pattern that resembled young muscle (Figure 2a,c). OXPHOS enzymes were also investigated by Western blot, which again as expected showed a marked increase in muscle of the PGC‐1α transgenic group (Figure 2b). Further analyses of markers previously associated with aging showed dramatic differences between the aged controls and PGC‐1α overexpressors. The 20S proteosome was decreased in the transgenic, whereas markers of autophagy, including beclin, LC3II/LC3I, and wip2 (and p‐wip2), were markedly increased. In addition, Pax 7, a marker of muscle progenitor satellite cells, was also substantially increased in the aged PGC‐1α transgenic muscle (Figure 3).

### 2.2 mtDNA alterations in aged muscle PGC‐1α overexpressor

We analyzed muscle mtDNA for age‐related abnormalities, such as copy number or levels of recombinant molecules. Using qPCR, we amplified different regions of the mouse mtDNA, including recombinant breakpoints, previously shown to accumulate in aging brain (Greaves & Turnbull, 2009). We also quantified a D‐loop repeat (CRM) identified in a model of aging, the mutator mouse with a proof‐reading defective polymerase gamma (Williams et al., 2010). The location of these regions is depicted in Figure 3c insert and panel d of Fig. S1. We found that the ratio of different mtDNA regions was not altered (Fig. S1A), demonstrating that mtDNA rearrangements were not present at high levels. When levels of mtDNA regions were normalized to a nuclear coded gene, muscle of PGC‐1α overexpressors showed higher levels of mtDNA, as expected (Fig. S1B). Finally, we directly quantified the levels of two reported age‐related mtDNA deletions (Neuhaus et al., 2014) by amplifying the deletion breakpoint. These deletion breakpoints (Del A and Del C) were indeed increased in aged muscle. However, the overexpression of PGC‐1α did not exacerbate this increase (Figure 3c).

### 2.3 Analyses of secreted myokines in aged PGC‐1α overexpressors

We tested whether known myokines were altered in sera of these mice. We used the multiplex myokine panel from Millipore, which includes the following: BDNF, Fractalkine/CX3CL1, Follistatin‐like Protein 1 (FSTL‐1), GDF8, IL‐15, Irisin, Osteocrin/Musclin, SPARC/Osteonectin, FGF‐21, IL‐6, and LIF. We could not detect above‐threshold signals for most of these molecules. We did detect signals for the following: Osteocrin/Musclin, SPARC/Osteonectin, FGF‐21, and IL‐6. Serum of aged PGC‐1α mice had mildly increased FGF21 and Osteocrin. No differences were detected for Osteonectin or IL‐6. Similar to most myokines tested, Irisin levels were below detection using the EMD reagents and detection curve standards. Lactate levels in serum were also determined by an enzyme assay and there was no difference between groups (Fig. S2).

### 2.4 PGC‐1α effect on the neuromuscular junction of aged muscle

**Figure 4:**
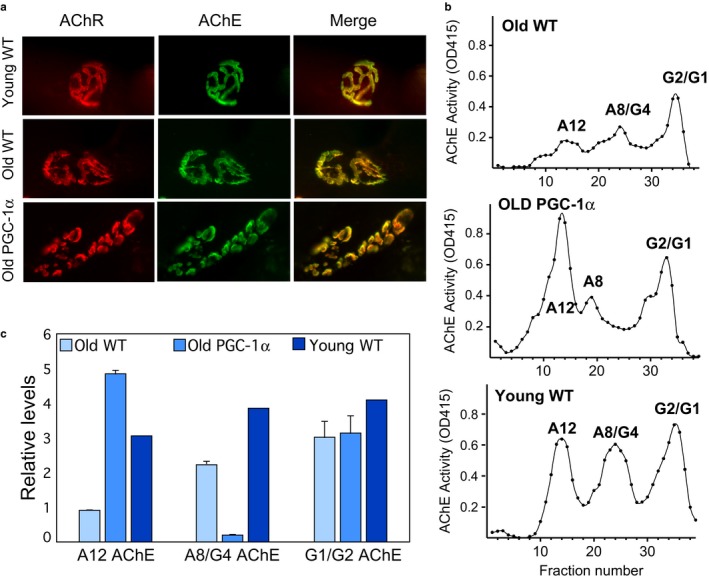
NMJ alteration during aging and in PGC‐1α overexpressor. Panel a shows NMJs stained with Alexa‐555 α‐bungarotoxin to label AChR and Alexa‐488 Fasciculin2 to label AChE to visualize the NMJ. Panel b shows sucrose gradient profiles of AChE activity, which separates the different oligomeric forms of AChE expressed in young and older animals. G1, G2, and G4 are the globular monomeric, dimeric, and tetrameric AChE forms, respectively. A8 and A12 indicate the positions of the asymmetric collagen‐tailed synaptic forms. Panel c shows the quantification of the different AChE forms. Graph represent the mean and error bars are *SEM*. *N* = 3 for the old samples (wt and PGC‐1α) and *N* = 2 for the young sample

PGC‐1α has been implicated in neuromuscular junction (NMJ) structure and function (Handschin et al., 2007). Therefore, we analyzed whether its expression in aged skeletal muscle altered NMJ organization and acetylcholinesterase (AChE) expression. For these experiments, we used muscle from mice that were 22 months old and young controls that were 3 months old. Older muscle (both wild‐type and MCK‐PGC‐1α) showed increased segmentation in NMJs, even though it was more evident in the PGC‐1α muscle (Figure 4). At the molecular level, there were significant changes in the pattern of AChE oligomeric forms expressed in the old WT muscle, with a decrease in the asymmetric A8 and A12 AChE forms (consisting of two to three tetramers of catalytic subunits, which are the major ones localized to the NMJ (Rotundo, 2003)). Remarkably, the pattern of AChE forms expressed in MCK‐PGC‐1α mice resembled the pattern of enzyme expressed in young mice muscles, with greater levels of A12 and an intermediate peak corresponding to asymmetric oligomeric forms of AChE. These findings suggest that increased expression of PGC‐1α in aging muscle preserves the NMJ molecular features and likely function in a younger state.

### 2.5 Transcriptional patterns in aged muscle PGC‐1α overexpressor

To further understand transcriptional changes triggered in aged muscle after a lifelong overexpression of PGC‐1α, we studied the transcriptome of quadriceps from aged PGC‐1α muscle overexpressors and controls by RNA‐Seq. This analysis identified 1,485 differentially regulated genes (*p* ≤ .05 and fold change ≥2), of which 885 and 600 were up‐ or downregulated, respectively (Table S2). The top over‐ and underexpressed genes were selected and ranked by a combination of *p*‐value <.05 and fold change ≥10 (Tables S3 and S4). These were mostly overexpressed genes, but it is unclear why their expression was highly altered in the PGC‐1α overexpressing muscle.

**Figure 5:**
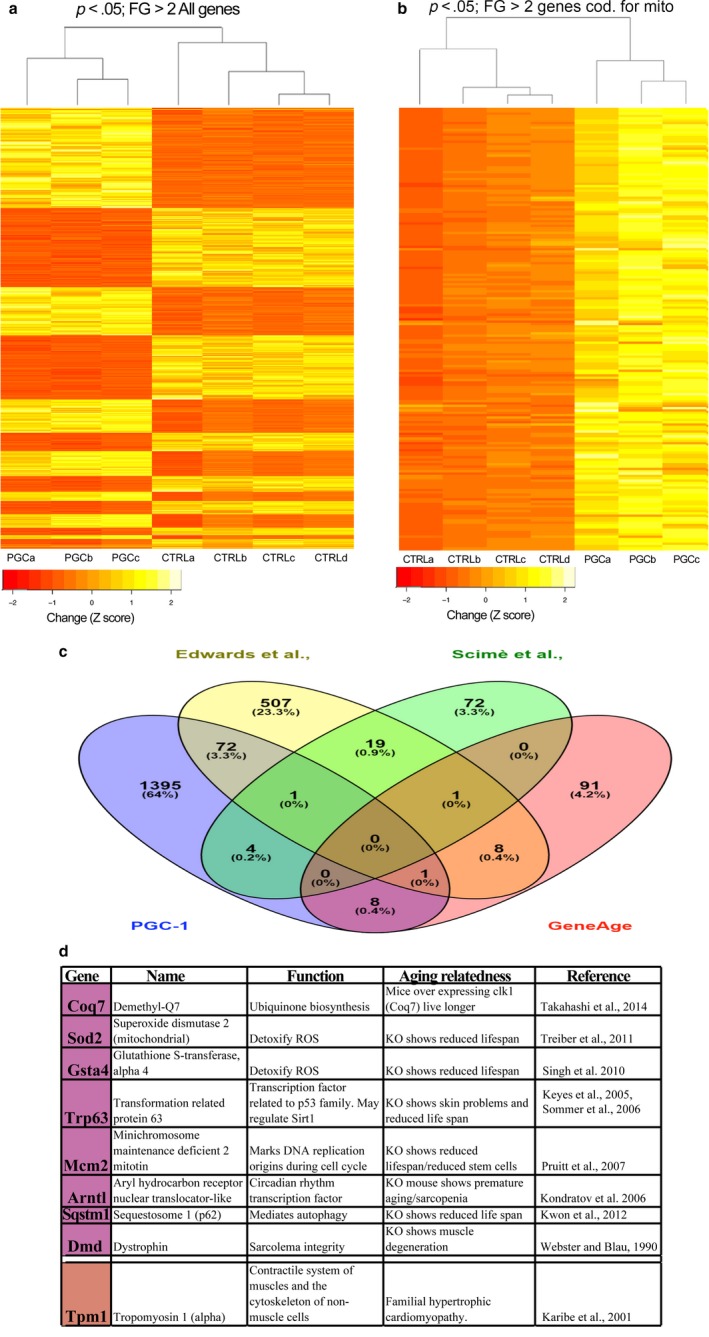
Transcriptome analyses of aged PGC‐1α vs. aged controls. Panels a–b show the clustering of the transcriptome pattern in aged muscle from controls and PGC‐1α overexpressors. Only genes with fold change larger than 2 and *p* < .05 were included in the analyses. Panel a shows all genes falling within these parameters, whereas panel b shows only genes coding for mitochondrial proteins. Yellow color indicates increased levels, whereas orange indicates decreased expression. The list of genes can be found in Table S2. Panel c shows a Venn diagram showing the overlap of genes altered in our study and genes altered in other age‐related mouse muscle studies, whereas panel d summarizes the function of the genes that specifically overlap with our PGC‐1α overexpressor and the GenAge dataset with potential relevance to longevity and/or aging

Initial global clustering analyses of all gene expression data showed that the PGC‐1α transgenic muscle has a very distinct pattern, clustering away from the control aged muscle (Figure 5a). When genes coding for mitochondrial proteins were analyzed separately, there was a clear upregulation of such genes in the muscle of the aged PGC‐1α transgenic mice, which still clustered away from control muscle (Figure 5b). We next performed a GO enrichment analysis that revealed statistically distribution of transcripts among GO categories in aged muscle overexpressing PGC‐1α. The overrepresented cellular components, biological processes, and molecular functions are displayed in Fig. S3 and showed a clear upregulation of genes related to energy metabolism and mitochondrial function. This analysis showed additional pathways that were altered, including antigen processing and presentation, and response to interferon‐gamma.

### 2.6 Transcriptional patterns altered in aged muscle PGC‐1α overexpressor with potential role in the aging process

We initially compared literature data with our data (at *p* < .05) and asked whether a subset of genes in aged muscle of PGC‐1α overexpressor overlap with data from studies that compared mouse young muscle with old muscle (Edwards et al., 2007; Scime et al., 2010) or with longevity genes from the GenAge mouse dataset (de Magalhaes & Costa, 2009). The intersection of all the four conditions tested showed no overlapping transcripts (Figure 5c). However, a total of 77 transcripts in our data overlap with the differentially expressed transcripts from one of the young muscle/old muscle studies (Figure 5c). Perhaps more relevant are genes that overlap between our PGC‐1α overexpressor and the GenAge dataset. The genes present in this database are well curated and therefore restricted to factors with demonstrated effects in lifespan or longevity, not survival. Interestingly, this analysis revealed the following genes altered in the aged PGC‐1α overexpressors with potential relevance to longevity and/or aging: Arntl, Dmd, Trp63 m, Mcm2, Sod2, Sqstm1, Coq7, and Gsta4. Figure 5d summarizes their function.

We next analyzed whether there were common pathways between our data and the young muscle/old muscle studies. Using the Reactome platform (http://www.reactome.org, Croft et al., 2011), we found that overexpression of PGC‐1α alters muscle contraction pathways that specifically overlap with these aging muscle studies (Table S5). This analysis also showed that the other two studies share five additional pathways, including integrin cell surface interactions, ECM proteoglycans, assembly of collagen fibrils, syndecan interactions, and translocation of GLUT4 to the plasma membrane.

### 2.7 Overexpression of PGC‐1α in skeletal muscle leads to a modest increase in lifespan in sedentary mice

**Figure 6:**
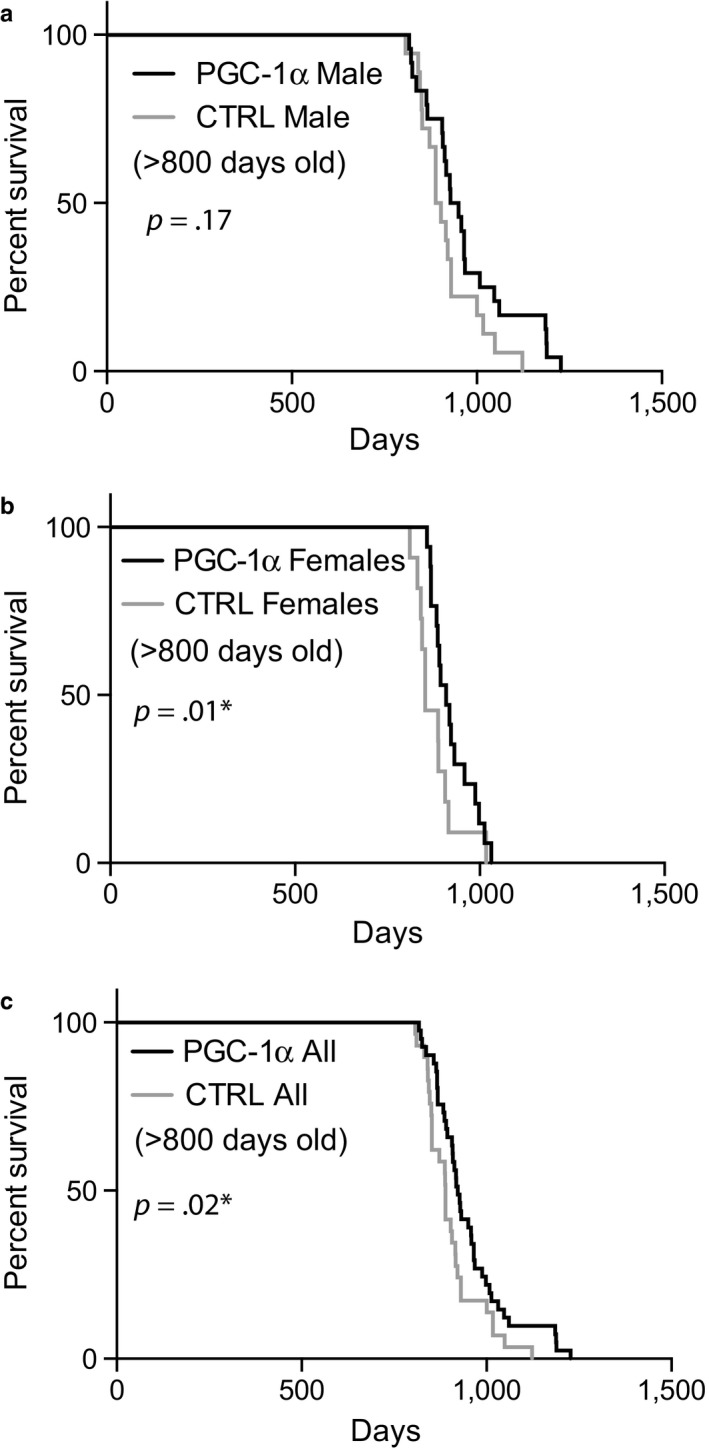
Kaplan–Meier survival curves of old mice overexpressing PGC‐1α in skeletal muscle. Two groups of mice that are either control C57/Bl6J or C57/Bl6J overexpressing PGC‐1α in skeletal muscle were analyzed for survival, *n* > 60 per group. We analyzed separately males (panel a), females (panel b), and combined sex (panel c). Although there was no difference between groups when all animals were analyzed (not shown), analyses restricted to mice that reached old age (more than 800 days) detected a modest extension (approximately 5%) in median lifespan (females and combined) and approximately 10% in maximal lifespan (males and combined)

Most of wild‐type and PGC‐1α muscle overexpressors (males and females) mice were allowed to age to “natural death” in standard vivarium conditions, with an average of three mice per cage. When total mortality was analyzed, no significant differences in median survival were found between the groups. However, when we analyzed mice that lived to old age (≥800 days), a statistically significant difference was observed, with an increase in median lifespan of approximately 5% (Figure 6). A statistical difference was found in the combined sex and in the female groups, whereas only a trend was observed in the male group (Figure 6). On the other hand, maximal lifespan (oldest 12% mice) was increased by approximately 10% in males but was not significantly changed in females.

## 3 DISCUSSION

PGC‐1α regulation of muscle function has been extensively studied (Correia, Ferreira, & Ruas, 2015). Several studies indicate that PGC‐1α at least partially mediates the benefits of exercise and caloric restriction (Kupr & Handschin, 2015). PGC‐1α regulation affects mitochondrial biogenesis, neuromuscular junction structure and organization, angiogenesis, ROS scavenging, fiber‐type balance to name a few critical aspects that could have an impact is muscle and organismal aging (Kupr & Handschin, 2015). We have previously shown that the transgenic overexpression of PGC‐1α in muscle improved some premature aging phenotypes in the Polg mutator mice (Dillon, Williams, et al., 2012). In the present study, we found a small but significant effect of increased muscle PGC‐1α on median lifespan in females and maximal lifespan in males when analyzing mice that reached old age.

Because the present study was initially designed exclusively to measure lifespan, we did not analyze muscle function during aging. However, muscle from these extremely old mice overexpressing PGC‐1α were analyzed and showed several molecular features that resembled muscle from younger mice. These include the expected increase in mitochondrial content, but also a rebalance of myosin heavy‐chain isoforms, higher levels of stem cell and autophagy markers, and lower levels of proteasome markers. These changes have been reported to occur in opposite directions during aging (Bilodeau, Coyne, & Wing, 2016; Blau, Cosgrove, & Ho, 2015). This suggests that the muscle of aged PGC‐1α overexpressors may have aged slower, at least in terms of the markers analyzed. Unfortunately, the levels of several know myokines were below detection using a multiplex system. There was a small increase in FGF21 and Osteocrin. FGF21 was shown to be increased in sera of humans with mtDNA‐related myopathies (Lehtonen et al., 2016). It is not clear why FGF21 levels would be increased in the PGC‐1α overexpressors, but it may be related to the fact that mitochondrial biogenesis is increased in both situations. Additional functional studies will be required to determine the benefits of the molecular changes described.

There were also remarkable changes in the composition of the NMJ during aging. We found that MCK‐PGC‐1α mice had NMJ that more closely resembles that observed in younger mice. Most interesting was the dramatic increase in the synaptic A12 AChE form in old PGC‐1α‐overexpressing mice compared to control aged mice (Figure 4), and again, closely resembling the ones from much younger mice, suggesting that the synapse may be physiologically “younger.” At the same time, there appears to be an increase in the normal fragmentation of the NMJ that occurs with age. While this has been observed by many investigators, it was only recently that an explanation for the fragmentation has been reported indicating that it is related to the extensive use and repair of the synaptic region in skeletal muscle (Li, Lee, & Thompson, 2011).

As PGC‐1α has a major role in controlling transcription, we analyzed the transcriptome in aged muscle by RNA‐Seq. These studies showed that hundreds of muscle transcripts were altered by the lifelong overexpression of PGC‐1α, with genes related to mitochondrial biogenesis forming the largest ontology group. However, several genes coding for nonmitochondrial proteins were also altered, some by more than 20‐fold. The group of transcripts overexpressed by more than 10‐fold did not cluster in specific pathways, but included genes coding for proteins associated with antioxidant regulation, metabolite transport, signal transduction, transcription, among several others. As functional groups, again there was a preponderance of change in pathways associated with energy metabolism, but also with antigen presentation. It is possible that these data reflect an increase in angiogenesis in PGC‐1α overexpression muscle, a previously established phenomenon (Kupr & Handschin, 2015).

The comparative gene ontology (GO) analyses identified nine genes with altered expression patterns that overlap between our PGC‐1α overexpressor and the GenAge database. This short list included: Arntl, Dmd, Trp63, Mcm2, Sod2, Sqstm1, Coq7, Gsta4, and Tpm1. Although diverse, these few genes may indicate a common tread between normal aging and adaptations triggered by PGC‐1α in muscle metabolism and regeneration. As shown in Figure 5d, all these factors are known to affect lifespan or muscle integrity, even if their anti‐aging mechanisms are not well understood. Several of these factors (Sod2, Coq7, and Gsta4) have been directly connected to production or elimination of ROS. Sod2 and Gsta4 eliminate superoxide and lipid peroxides, respectively (Hubatsch, Ridderstrom, & Mannervik, 1998), whereas coenzyme Q10 redox reactions lead to the production of superoxide in the context of complex III (Acosta et al., 2016). Coq7, also known as Clk1, is essential for life, but reducing its levels led to increased lifespan in mice (Takahashi, Noda, Ohsawa, Shirasawa, & Takahashi, 2014). Sqstm1, also known as p62, is a mediator of autophagy, but also mitochondrial dynamics, import, and genome integrity (Seibenhener et al., 2013). Arnt1 and Trp63 are transcription factors. Arnt1 has been shown to regulate mymesin 2, a cytoskeletal protein in skeletal muscle and heart (Woods, Farrall, Procko, & Whitelaw, 2008), whereas Trp63 regulates, among other processes, late stages of myogenesis (Cefalu et al., 2015). Mcm2 marks the nuclear DNA during replication (O'Donnell & Li, 2016) and it is downregulated during muscle differentiation (Kislinger et al., 2005). Dmd, the affected gene in Duchene muscular dystrophy, is part of the protein complex conferring sarcolemma integrity (Matsumura et al., 1993). Interestingly, PGC‐1α overexpression in muscle has been shown to be protective in Dmd‐deficient mice (Chan et al., 2014).

Noteworthy, the two previous studies comparing C57/BL6 mice (young vs. old) showed very few differentially expressed genes in common. Our data from old mice overexpressing PGC‐1α had 74 genes in common with one study (Edwards et al., 2007) and five genes with the other (Scime et al., 2010). There was only one gene in which expression was altered in our study, the GenAge database and one of the “young vs. old” mouse muscle study (Edwards et al., 2007), the tropomyosin 1 (Tpm1) transcript. Tpm1 is a versatile gene and encodes at least 10 tissue‐specific variants via alternative splicing and/or the use of two promoters (Denz, Narshi, Zajdel, & Dube, 2004). TPM1 is also a tumor suppressor gene and more recently was found to influence the age of onset in Parkinson's disease by 15 years (Hill‐Burns et al., 2016).

Also curiously, these two studies did not identify a large number of genes coding for mitochondrial proteins with altered expression with C57BL6 aging. This is in dramatic contrast with studies performed in human muscle, which showed that this functional group (oxidative phosphorylation) was the most affected GO group affected in aging muscle (Liu et al., 2013). In fact, in a recent study, 455 of 12,044 genes were differentially expressed in aging muscle and heart and approximately 44% of these genes were associated with the Krebs cycle and OXPHOS (Seim, Ma, & Gladyshev, 2016). Studies in Drosophila thorax showed a similar pattern, with mitochondrial‐related genes being the most prominent functional group altered with aging (Girardot, Lasbleiz, Monnier, & Tricoire, 2006). We found the same common pattern (OXPHOS altered) between our PGC‐1α data and in the C57BL6 muscle published studies (Edwards et al., 2007; Scime et al., 2010), but only if using a less strict p value for the previously published studies (below statistical significance). At this point, we cannot assess the validity of the previous studies on C57BL6 muscle transcriptome of aged mice, but we suspect that mitochondrial‐associated genes are grossly under‐represented in these studies.

In conclusion, we found that lifelong overexpression (approximately sixfold) of PGC‐1α in muscle was associated with a modest extension in lifespan. However, muscle of these very old mice showed marked differences in their morphology, structural composition, proteome, and transcriptome. These include not only an increase in energy metabolism but also adaptations in pathways associated with oxidation, muscle regeneration, and remodeling. We conclude that although the “longevity” effects of PGC‐1α muscle overexpression may be modest, muscle itself is remodeled to better resemble younger muscle at the molecular and cellular levels. Although overexpression of PGC‐1α is not a feasible approach for humans, several groups are currently screening for small molecules to enhance the mitochondrial biogenesis cascade.

## 4 EXPERIMENTAL PROCEDURES

### 4.1 Animal husbandry

The mice were kept in a condition of 12‐hr light/dark cycle at room temperature. They were fed a regular diet (Rodent Chow 5010, Harlan) and were kept at 3–4 mice per cage. Mice were allowed to age until natural death or developed conditions that required DVR‐mandated termination. Males’ PGC‐1α [*n* = 40], females’ PGC‐1α [*n* = 38], male controls [*n* = 26], female controls [*n* = 28].

### 4.2 Histochemistry and immunohistochemistry

Muscle tissue was frozen in isopentane liquid nitrogen. Cross sections (8 μm) were stained for COX, SDH, and combined activities. Muscle sections were also stained with H&E to assess muscle fiber area and general morphology. For immunohistochemistry, muscle sections were treated with 0.5% Triton X‐100 in PBS for 30 min and washed in PBS for 30 min before the addition of primary antibodies (AbCam).

### 4.3 Western blots

Western blot analysis was performed as described previously (Dillon, Williams, et al., 2012). Antibodies against different subunits of the oxidative phosphorylation complexes and VDAC were obtained from AbCam. Antibodies against muscle proteins were obtained from the Developmental Studies Hybridoma Bank at the University of Iowa.

### 4.4 Mitochondrial DNA analyses

Muscle total DNA was analyzed by quantitative real‐time PCRs in the presence of a fluorescent dye (SYBR Green, QIAGEN) as described (Dillon, Williams et al., 2012). The results were normalized to the nuclear β‐actin gene. The primers used for CRM are described in Williams et al. (2010) and the ones for age‐related deletions in (Neuhaus et al., 2014). The remaining primers are listed in Table S6.

### 4.5 Multiplex myokines analyses

Blood was withdrawn from deeply anesthetized animals by cardiac puncture. To analyze the levels of mouse myokines in mouse plasma, we used the mouse myokine panel from Millipore (MMYOMAG‐74K**,** Luminex) and followed the Millipore protocol described in: http://www.emdmillipore.com/US/en/product/MILLIPLEX-MAP-Mouse-Myokine-Magnetic-Bead-Panel,MM_NF-MMYOMAG-74K. The myokines included the following: BDNF, Fractalkine/CX3CL1, Follistatin‐like Protein 1 (FSTL‐1), GDF8, IL‐15, Irisin, and LIF

### 4.6 Analysis of the neuromuscular junction

AChE enzyme activity and oligomeric forms were measured as described (Rossi & Rotundo, 1993). Briefly, hind limb muscles were homogenized in 10 volumes of extraction buffer consisting of 20 mm sodium borate buffer, pH 9.0, 1 m NaCl, 1.0% Triton X‐100, 10 mm EDTA, and protease inhibitors. Aliquots were analyzed by velocity sedimentation on 5%–20% sucrose gradients and the fractions assayed for AChE activity using the colorimetric Ellman assay (Rossi & Rotundo, 1993). For analysis of AChE and AChR, the images were captured using identical exposure parameters and the fluorescence intensity at each wavelength expressed as relative units. For quantitative analysis of the neuromuscular junctions, mouse thigh muscles were labeled with Alexa‐488 Fasciculin2 and Alexa‐555α‐Btx. Digital images were taken at two wavelengths appropriate to each fluorophore using a Princeton Instruments Micromax camera mounted on a Leica DMR‐A microscope. Images were captured using Slidebook 4.0 and quantified using a calibrated optical micrometer.

### 4.7 Statistics

Two‐tailed, unpaired Student's *t*‐test was used to determine the statistical significance between the different groups. If more than two groups were analyzed, significance of the differences was evaluated by one‐way ANOVA followed by Bonferroni post‐test. Survival data were analyzed by Prism 6 for Mac (GraphPad Software) applying Kaplan–Meier curves and Gehan–Breslow–Wilcoxon test.

### 4.8 Deep transcriptome sequencing (RNA‐Seq)

Total RNA (5 μg) was used to construct RNA‐Seq libraries for CTRL (*n* = 4) and PGC‐1α (*n* = 3) groups that were sequenced in a same flow cell, as paired‐end, 2 × 100 bp, on an Illumina HiScanSQ instrument (Illumina, USA), following manufacturer's instructions, which generated an average of 24 paired‐end reads per sample (submitted to the SRA under accession PRJEB15213). The raw sequence files (.fastq files) underwent quality control analysis using FastQC, and average Phred quality scores of ≥26 per position were used for alignment. Reads were aligned to the reference mouse genome (RefSeq, mm9) using Burrows–Wheeler Alignment (BWA; Li & Durbin, 2009), and then the number of reads per gene was counted using htseq‐count. Differential expression analysis across CTRL and PGC‐1α groups were performed using DEseq algorithm, R package (version 1.22.1) and reported as fold change along with associated *p*‐values. Cutoffs for significant changes were a fold change >1.5 and a *p*‐value ≤.05. Finally, Zscore normalization was used for visualization purposes. The analyses were performed using the R Bioconductor packages ggplot2 (version 2.1.0), gplots (version 3.0.1), heatmap (version 3.2.5), and heatmap.2 (version 3.0.1) on the programming language r (version 3.2.5).

### 4.9 Gene ontology enrichment analysis

To identify the overrepresented GO categories of differential expressed genes, we performed the cellular components, biological processes, and molecular functions enrichment analysis available from PANTHER classification system (Mi, Muruganujan, Casagrande, & Thomas, 2013; Mi, Poudel, Muruganujan, Casagrande, & Thomas, 2016) found at http://www.pantherdb.org/. The GO categories with *p*‐value ≤.05 were considered significant.

### 4.10 Reactome analyses

Data were analyzed at http://www.reactome.org as instructed (version 57). Pathway Analysis tools analyze user‐supplied datasets permitting ID mapping, pathway assignment, and overrepresentation or enrichment analysis (Fabregat et al., 2016).

## CONFLICT OF INTEREST

None declared.

## AUTHOR CONTRIBUTIONS

Sofia Garcia participated in molecular experiments; Nadee Nissanka participated in molecular experiments and edited manuscript; Edson Mareco was involved in gene expression analyses; Susana Rossi was involved in NMJ experiments, prepared figures, and edited manuscript; Susana Peralta contributed to real‐time PCR and Western blots; Francisca Diaz contributed to myokine experiments and analyses, and edited manuscript; Richard L. Rotundo contributed to NMJ analyses and edited manuscript; Robson Carvalho contributed to gene expression experiments and analyses, planned experiments, and wrote manuscript; Carlos T. Moraes was involved in the longevity and molecular analyses, planned experiments, and wrote manuscript.
