# Supplementary material for: Overexpression of PGC‐1α in aging muscle enhances a subset of young‐like molecular patterns
Source: Aging Cell. 2018 Feb 10;17(2):e12707. doi: 10.1111/acel.12707 (PMC5847875; doi:10.1111/acel.12707)
Supplement: Supplementary file 1 [file ACEL-17-e12707-s001.pdf]

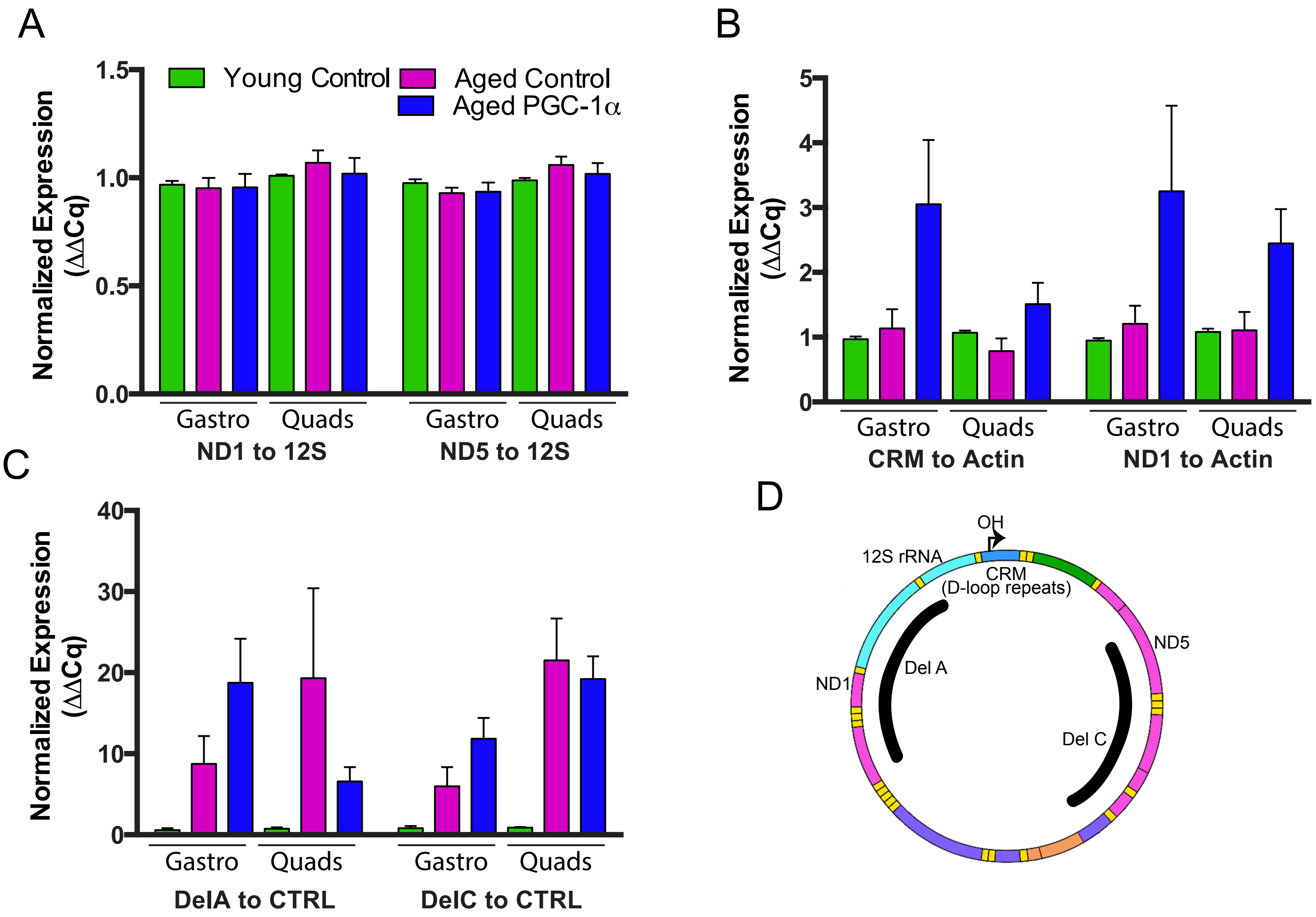

Supplemental Figure SF1. Effect of PGC-1α overexpression in the accumulation of age-associated mtDNA deletions.

Ratios of ND1/12S and ND5/12S genes, determined by qPCR, were not altered in aged muscle (Gastro and Quads) overexpressing PGC-1α (Panel A). Steady-state levels of mtDNA (ND1/actin) also measured by qPCR were increased in PGC-1α overexpressing muscles (panel B). The D-Loop region was equally increased (Panel B), suggesting that it was not particularly expanded. Previously reported age-related deletions (known as Deletion A and Deletion C) were increased in aged muscle (measured by qPCR), but no different between the groups (Panel C). Panel D shows the location of the deletions, which were first reported in (Neuhaus et al. 2014). Panel C is the same as panel C in Fig.3.

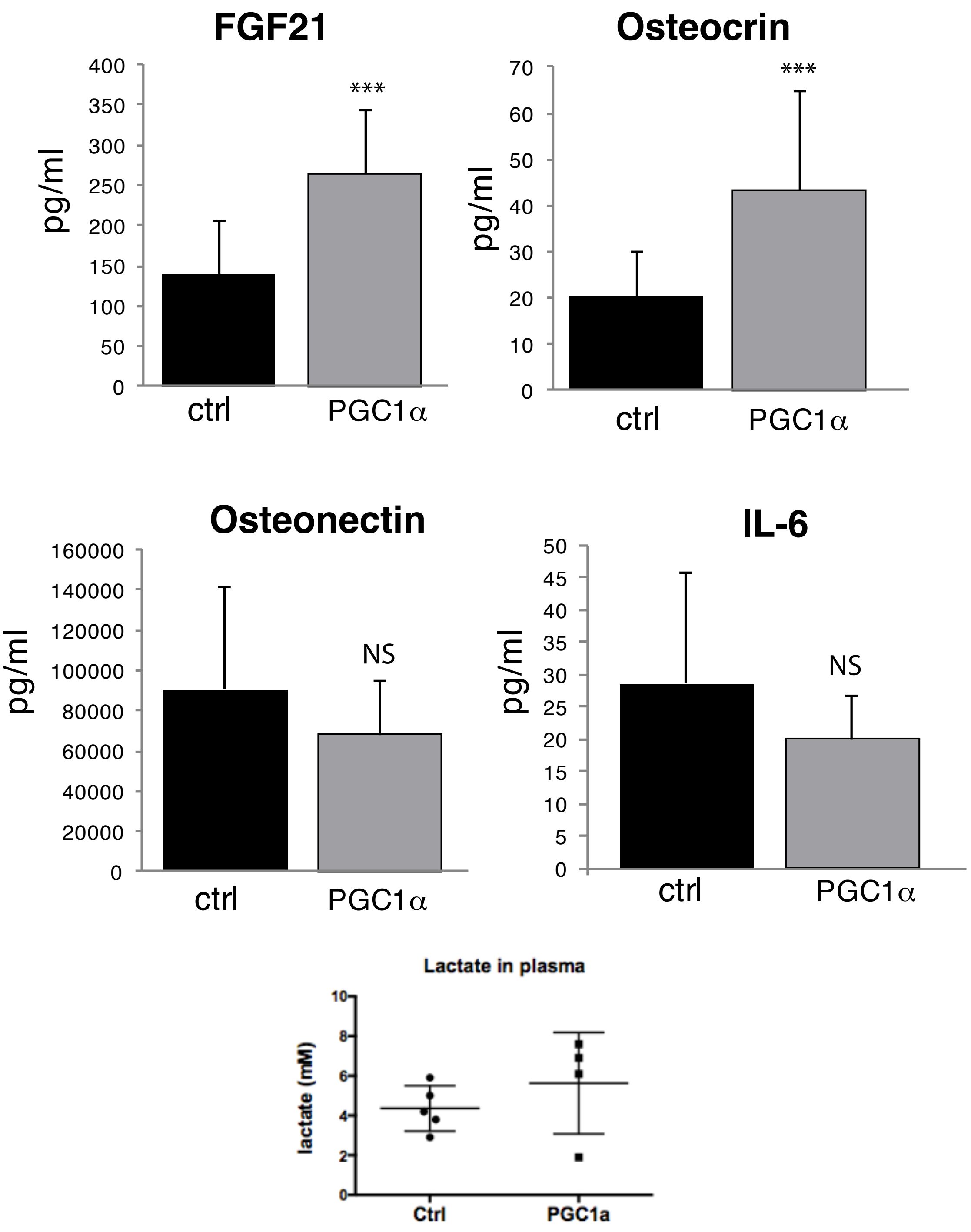

Supplemental Figure SF2. Myokine analyses in old mice overexpressing PGC-1α. Of the 11 myokines analyzed, only 4 were above detection limits. These were: FGF21, Osteocrin,Osteonectin and IL-6. Only FGF21 and Osteocrin were altered in PGC-1α mice. Lactate was also analyzed and not found to be different between groups.

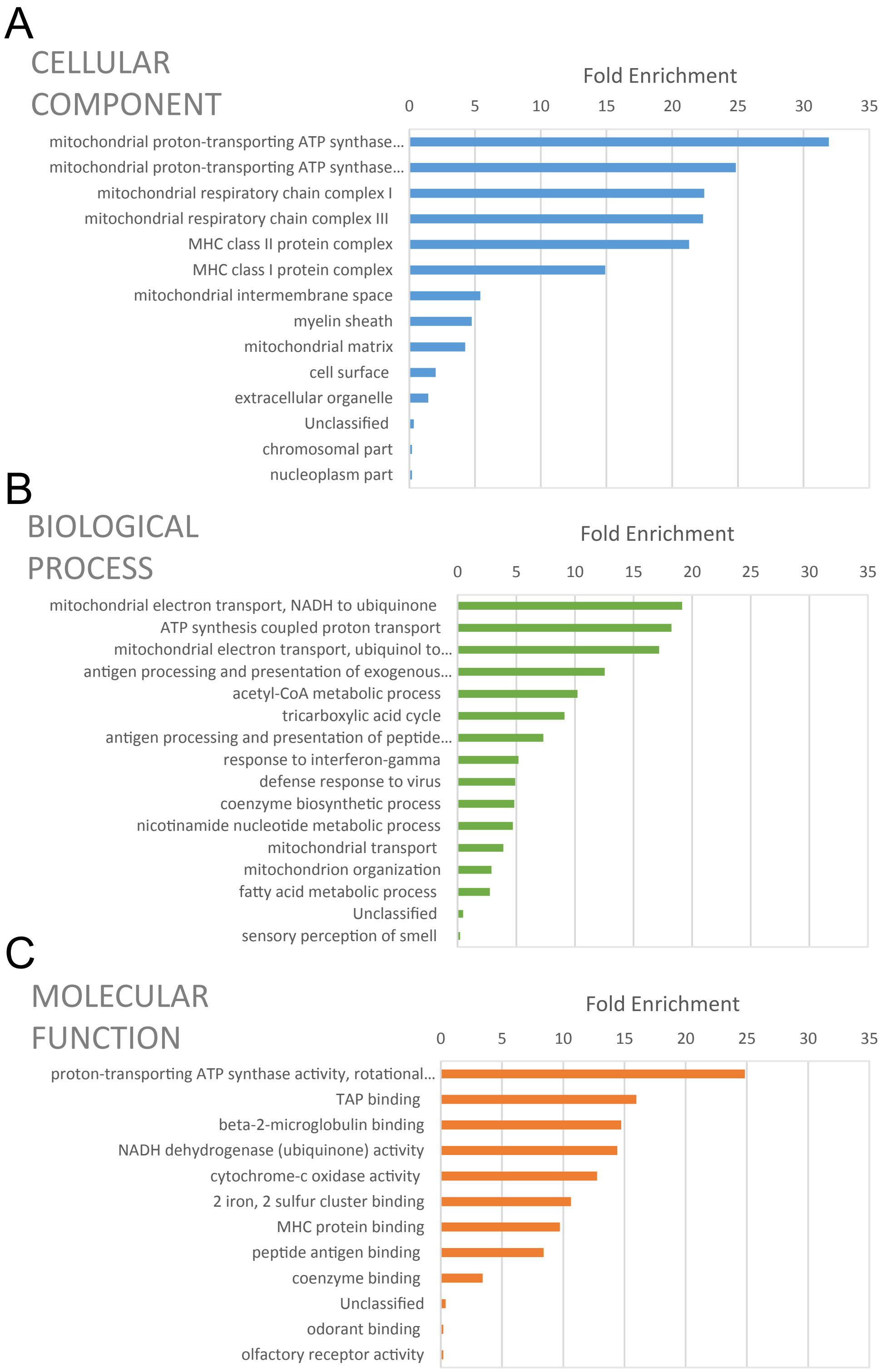

Supplemental Figure SF3. Gene Ontology analyses of aged mouse muscle over expressing PGC-1α. The figure shows the functional groups over represented in our data set. Panel A shows the fold-enrichment of gene products grouped by Cellular Component, Panel B by Biological Process, and Panel C by Molecular Function.
