# Supplementary material for: Overexpression of PGC‐1α in aging muscle enhances a subset of young‐like molecular patterns
Source: Aging Cell. 2018 Feb 10;17(2):e12707. doi: 10.1111/acel.12707 (PMC5847875; doi:10.1111/acel.12707)
Supplement: Supplementary file 2 [file ACEL-17-e12707-s002.pdf]

Supplemental Table ST1. Mice used for molecular studies.

Mouse ID	Sex	Genotype	Age
6Bp3m18	F	PGC-1α	868
6Bp3m19	F	PGC-1α	868
122210m19	M	PGC-1α	755
010611m2	M	PGC-1α	1008
122810m6	F	CTRL	1017
6Bf3m18	M	CTRL	889
6Bf3m17	M	CTRL	889
6Bf3m19	M	CTRL	889
122810m3	M	CTRL	1017
